# Granular cell tumor presenting in the scrotum of a pediatric patient: a case report and review of the literature

**DOI:** 10.1186/s13256-016-0911-x

**Published:** 2016-06-04

**Authors:** Abby M. Richmond, Francisco G. La Rosa, Sherif Said

**Affiliations:** 1 Department of Pathology, University of Colorado Anschutz Medical Campus, Aurora, CO 80045 USA; 2 Department of Pathology, Denver Health Hospital, Denver, CO 80204 USA

**Keywords:** Granular cell tumor, Scrotum, Pediatric, Pathology

## Abstract

**Background:**

Granular cell tumors are neoplasms of Schwann cell origin. They typically arise in the head and neck of adults, with the tongue being the most common location; granular cell tumors of male genitalia are exceedingly rare. We identified only eight prior cases of scrotal granular cell tumor in the literature, and only one was in a child. Herein, we report a second case of childhood scrotal granular cell tumor and provide a review of the most relevant literature.

**Case presentation:**

A fifteen-year-old hispanic boy was referred to our hospital's pediatric surgery service for a painless and firm scrotal mass. Clinical impression was that of an epidermal inclusion cyst. There was no evidence of associated medical problems from the clinical history and physical examination. Surgical enucleation of the lesion demonstrated a solid nodule with morphological and immunohistochemical features consistent with a benign granular cell tumor.

**Conclusions:**

This is the second case reported of a scrotal granular cell tumor in a child. Although genital granular cell tumors are rare, and most are benign, careful clinical examination, complete surgical excision, expert histologic evaluation, and a close follow-up are recommended for accurate diagnosis and to rule out eventual malignancy.

## Background

Granular cell tumors (GCT) are soft tissue neoplasms, also known as Abrikossoff tumors, which have a neural origin derived from Schwann cells [1, 2]. GCT are most often located in the dermis or subcutaneous tissue, and less frequently in the submucosa, smooth muscle, and striated muscle. The fine granular aspect of the tumor cells is due to the accumulation of lysosomes in the cytoplasm, which may be also observed in many tumors with no neuronal origin, such as smooth muscle, connective tissue, neuroglia, endothelial, and epithelial cells [1, 3]. Hence, expert pathological evaluation is required for establishing the diagnosis of these lesions. If examined with routine light microscopy, GCT may appear indistinguishable from other tumors with granular cell morphology, including basal cell carcinoma, melanoma, leiomyoma, leiomyosarcoma, dermatofibrosarcoma, angiosarcoma, fibrous histiocytoma, and ameloblastoma. Thus, the use of additional immunohistochemical stains is critical for the ultimate identification of their neural phenotype. GCT are positive for S-100 protein, myelin proteins, and myelin-associated glycoprotein, and are in general negative for epithelial, melanocytic, smooth muscle, dendritic cell, and endothelial markers [1, 2]. The differential diagnosis may also include rhabdomyoma, which are desmin and myoglobin positive, and granular cell histiocytic reactions [1]. GCT cells may be also positive for NKI-C3 (CD63), NSE, CD68, calretinin, inhibin-alpha, protein gene product 9.5 (PGP. 9.5), nestin, and low-affinity nerve growth factor receptor (p75). Melan-A may be focally positive and microphthalmia transcription factor (MITF) is often diffusely positive, but HMB45, cytokeratin, epithelial membrane antigen, and desmin are consistently negative [1, 4].

The incidence of GCT is rare, usually reported in the literature as single cases, with a predominant location in the skin of the head and neck, and most of them are located in the oral cavity (70 %), the tongue being the most common site; they also have been described in the breast and in some internal organs in the upper respiratory and gastrointestinal tracts. The typical presentation of GCT is that of a solitary painless nodule in which blacks are more commonly affected than whites, with a peak incidence in the fourth to sixth decades, and with women affected slightly more often than men (female-to-male ratio is near 3:2) [3]. Multiple GCT may be seen in the context of LEOPARD syndrome (Noonan Syndrome), a rare autosomal-dominant, multisystem disease caused by a mutation in the protein tyrosine phosphatase, nonreceptor type 11 gene (PTPN11) [5]. Several reports have also suggested some association between multiple cutaneous GCT and neurofibromatosis, or elements of this disease [6], but the small number of cases reported are not sufficient to demonstrate a true association between these two entities. Therefore, in the presence of a nodular lesion, a very careful and complete clinical examination is recommended to rule out underlying somatic and genetic syndromes.

Most GCTs are benign, nonulcerated and usually painless nodules, with an insidious onset and slow growth rate, in which complete surgical excision is usually curative. Nevertheless, the recurrence rates of benign lesions are 2–8 %, even when the resection margins show no evidence of tumor; disease recurrence may be near 20 % when the margins are positive for tumor. Malignant cases are rare and represent 1–2 % of cases; they are usually larger (>5 cm) and may be locally destructive, causing symptoms of pressure, obstruction, hemorrhage, ulceration, and/or secondary infection, and demonstrating rapid growth, local recurrence, and distant metastases [3]. Malignant lesions are very difficult to eradicate even with en bloc surgery. The local recurrences are >30 %, and metastatic disease may be present within 2 years in half of the cases, with approximately 40 % mortality within 3 years of tumor diagnosis [3].

Scrotal neoplasms in children are rare and mostly represented by benign tumors of skin and adnexal structures. Cases of pediatric GCT are extremely rare, with our case being the second one reported in the literature [7]. Thus, careful clinical examination of scrotal masses with complete surgical excision, followed by expert histologic examination, is essential to rule out rare malignant counterparts of other more common benign entities.

## Case presentation

**Fig. 1:**
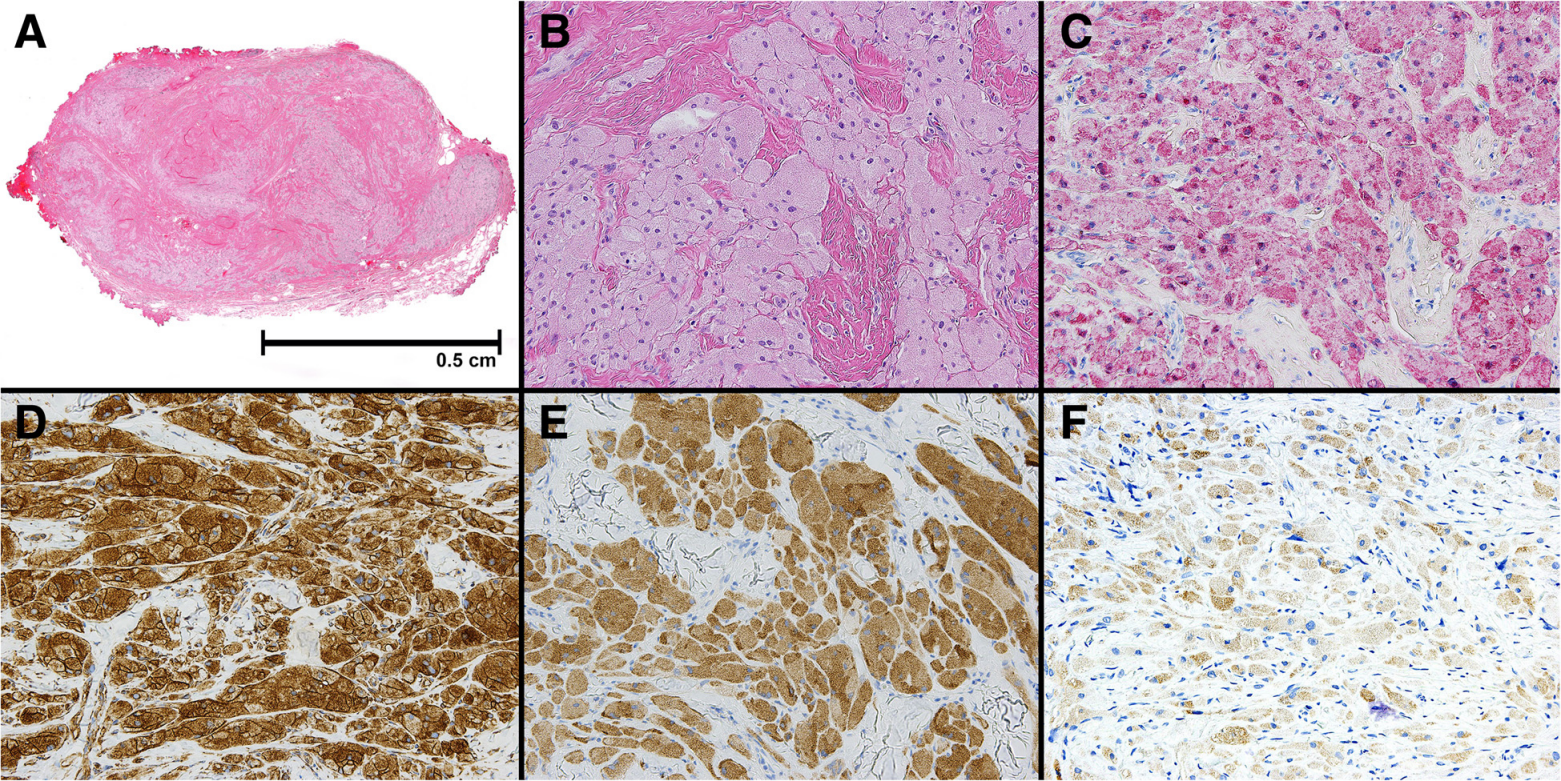
Representative photomicrographs of the lesion. Hematoxylin and eosin staining shows in (**a**) a poorly circumscribed nodular lesion arising within dermal and subcutaneous tissue that appeared completely excised (1× objective); in (**b**) the lesion is composed of a proliferation of polyhedral cells with abundant granular cytoplasm and small, uniform, dark nuclei, arranged in nests, and single cells surrounded by fibrous connective tissue. Immunoperoxidase staining shows in (**c**) the tumor cells positive (3+) for S-100 staining (alkaline phosphatase substrate), (**d**) positive (3+) for vimentin, (**e**) positive (2+) for neuron-specific enolase and (**f**) positive (1+) for inhibin-A (**d**-**f**, horseradish peroxidase substrate) (**b**-**f** 20× objective)

A 15-year-old boy of Hispanic origin with an unremarkable past medical history was noted to have a scrotal nodule during a routine physical examination. No additional mucosal or cutaneous lesions were identified elsewhere. Our patient recalled that the nodule had been present for 3–4 years, it was nonpainful, and with no significant growth. A clinical examination of his scrotum revealed a firm, nodular lesion present immediately under a yellowish, discolored skin. Our patient was referred to pediatric surgery where he had complete surgical enucleation of the nodular lesion with no further complications. Gross examination of the excised specimen revealed a 1 × 0.8 × 0.6 cm firm, nonencapsulated mass covered by loose fibrous tissue; no evidence of associated skin tissue was observed. Sectioning of the nodule revealed a whitish, homogenous cut surface with no evidence of hemorrhage or necrosis. No photographic record of the gross specimen is available, however, Fig. 1a shows a low-power histological image of the hematoxylin and eosin staining of a whole section of the lesion, which appears to be completely excised with no involvement of the surgical margins. Microscopic examination at higher power demonstrated a poorly circumscribed proliferation of polyhedral cells with abundant granular cytoplasm and small, uniform, dark nuclei (Fig. 1b). The lesional cells were present within fibrous connective tissue as nests and individual cells. Positive staining for S-100 (Fig. 1c), vimentin (Fig. 1d), neuron-specific enolase (Fig. 1e), and inhibin-A (Fig. 1f) confirmed the diagnosis of a benign granular cell tumor [3]. At the time of this publication our patient has no evidence of disease recurrence or any other surgery-associated complication.

## Discussion

This report represents the ninth case of scrotal granular cell tumor and the second such case in a pediatric patient. The characteristics of this case and the previously described cases are summarized in Table 1.

**Table 1:** Bibliographic summary of granular cell tumors of the scrotum reported in the literature

Case	report	Age (years)	Race	Tumors	Size (cm)	Presentation	Treatment	Outcome
1	Bryant (1995) [9]	36	White	1	0.4	Painless nodule	Excision	Disease-free (6 months)
						Present for 1 year		
						No recent growth		
2	Altman (1999) [10]	55	Black	2	0.7, 1.1	Painless nodule	Excision	Disease-free (22 months)
3	Medina Perez (1999) [11]	19	N/A	1	2.0	Ulcerated skin	Excision	Disease-free (24 months)
						Present for months		
4	Menendez Lopez (2001) [12]	38	N/A	1	1.0	Painless nodule	Excision	N/A
						Present for 2 years		
5	Craig (2005) [13]	67	Black	1	1.6	Painless nodule	Biopsy, excision	N/A
						Present for 2 years		
						Recent enlargement		
6	Godoy (2008) [7]	57	White	1	1.5	Painless nodule	Biopsy, excision	Disease-free (6 months)
						Present for 35 years		
						Recent enlargement		
7	Sidwell (2008) [14]	6	N/A	1	0.5	Painless nodule	Excision	N/A
						Present for few months		
						Recent enlargement		
8	Chen (2013) [15]	89	Asian	1	1.5	Painless nodule	Excision	N/A
						Present for 3 weeks		
						No recent enlargement		
9	Present case	15	Hispanic	1	1.0	Painless nodule	Excision	Disease-free (1 month)
						Present 3–4 years		
						No recent enlargement		

*N/A* not available

Patients who present to the consult with a nodular lesion, regardless of location, should receive a very careful physical examination to rule out underlying somatic and genetic syndromes. After complete surgical excision, the histological criteria for the identification of malignancy are poorly defined, since cases with only mild atypia have metastasized. However, features that should raise the possibility of malignancy include a large tumor size (>5 cm), as well as necrosis, spindling, and vesicular nuclei with large nucleoli [3]. Similar to benign GCT arising in other parts of the body, benign scrotal GCT typically present as solitary painless nodules and are successfully treated with complete excision. There have been no reported cases of malignant scrotal GCT, but even so, excision and histologic examination is recommended to confirm a benign diagnosis. Benign GCT are characterized by ill-defined nodules within dermal and subcutaneous tissue, typically less than 3 cm. The overlying skin frequently demonstrates pseudoepitheliomatous hyperplasia, which could not be recognized in our case since no skin was submitted with the nodular lesion. The tumor cells demonstrate nested architecture separated by fibrous connective tissue septae. Individual cells are round to polygonal with small dark nuclei surrounded by abundant granular cytoplasm. The cytoplasmic granularity is attributed to the presence of histiocytes (CD68-positive) with PAS-D-positive lysosomes [2]. Neural origin is evidenced by frequent proximity to peripheral nerves and immunoreactivity to S-100, myelin proteins, and neuron-specific enolase. Recent studies show that GCT are also immunoreactive to inhibin, for unknown reasons [1, 4]. Although malignant GCT are defined by their metastatic behavior, the histologic criteria proposed by Fanburg-Smith *et al.* provide some guidance to identify high-risk cases. The presence of three of the following features indicates malignancy: necrosis, spindling, vesicular nuclei with large nucleoli, increased mitotic activity, high nuclear to cytoplasmic ratio, and pleomorphism [3]. This case demonstrated none of the aforementioned features. Only one prior case of scrotal GCT reported atypical histology on shave biopsy, but the features of malignancy were not mentioned in the resection specimen [7]. Appropriate pathologic examination is of utmost importance to identify high-risk features, as management differs for benign and malignant GCT. This is because of the higher recurrence rate and mortality in the latter, 32 % and 50 %, respectively [3]. In contrast to benign GCT, malignant GCT tend to infiltrate surrounding tissues and should be treated with wide local excision and regional lymph node dissection. Unfortunately, chemoradiation has not been shown to improve outcome [8].

## Conclusions

This case highlights the importance of excision and histologic examination of scrotal masses for accurate diagnosis and appropriate follow-up. GCT of the scrotum are rare, and thus far all reported cases have been benign. Nevertheless, adequate evaluation is advised to rule out possible malignancy.
